# Aligner optimization increases accuracy and decreases compute times in multi-species sequence data

**DOI:** 10.1099/mgen.0.000122

**Published:** 2017-07-08

**Authors:** Kelly M. Robinson, Aziah S. Hawkins, Ivette Santana-Cruz, Ricky S. Adkins, Amol C. Shetty, Sushma Nagaraj, Lisa Sadzewicz, Luke J. Tallon, David A. Rasko, Claire M. Fraser, Anup Mahurkar, Joana C. Silva, Julie C. Dunning Hotopp

**Affiliations:** ^1^​ Institute for Genome Sciences, University of Maryland School of Medicine, Baltimore, MD, USA; ^2^​ Department of Microbiology and Immunology, University of Maryland School of Medicine, Baltimore, MD, USA; ^3^​ Department of Medicine, University of Maryland School of Medicine, Baltimore, MD, USA

**Keywords:** genome sequence alignment, BWA, dual-species alignment, *Plasmodium*, *Brugia*, *Wolbachia*

## Abstract

As sequencing technologies have evolved, the tools to analyze these sequences have made similar advances. However, for multi-species samples, we observed important and adverse differences in alignment specificity and computation time for bwa- mem (Burrows–Wheeler aligner-maximum exact matches) relative to bwa-aln. Therefore, we sought to optimize bwa-mem for alignment of data from multi-species samples in order to reduce alignment time and increase the specificity of alignments. In the multi-species cases examined, there was one majority member (i.e. *Plasmodium falciparum* or *Brugia malayi*) and one minority member (i.e. human or the *Wolbachia* endosymbiont *w*Bm) of the sequence data. Increasing bwa-mem seed length from the default value reduced the number of read pairs from the majority sequence member that incorrectly aligned to the reference genome of the minority sequence member. Combining both source genomes into a single reference genome increased the specificity of mapping, while also reducing the central processing unit (CPU) time. In *Plasmodium*, at a seed length of 18 nt, 24.1 % of reads mapped to the human genome using 1.7±0.1 CPU hours, while 83.6 % of reads mapped to the *Plasmodium* genome using 0.2±0.0 CPU hours (total: 107.7 % reads mapping; in 1.9±0.1 CPU hours). In contrast, 97.1 % of the reads mapped to a combined *Plasmodium–*human reference in only 0.7±0.0 CPU hours. Overall, the results suggest that combining all references into a single reference database and using a 23 nt seed length reduces the computational time, while maximizing specificity. Similar results were found for simulated sequence reads from a mock metagenomic data set. We found similar improvements to computation time in a publicly available human-only data set.

## Abbreviations

BWA, Burrows–Wheeler aligner; CPU, central processing unit; LGT, lateral gene transfer; MEM, maximum exact matches; SRA, Sequence Read Archive; SW, Smith–Waterman Alignment.

## Data Summary

The human dataset analyzed during the current study is available from the 1000 Genomes Project in the Sequence Read Archive (SRA) (http://www.internationalgenome.org, accession number ERR022446), the three *Plasmodium–*human datasets are available from the Wellcome Trust Sanger Institute in the SRA (accession numbers ERR015379 https://www.ncbi.nlm.nih.gov/sra/?term=ERR015379, ERR012739 https://www.ncbi.nlm.nih.gov/sra/?term=ERR012739, and ERR0153 60 https://www.ncbi.nlm.nih.gov/sra/?term=ERR015360), the *Brugia–Wolbachia* dataset is available from the University of Maryland Institute for Genome Sciences in the SRA (accession number SRR5188379 https://www.ncbi.nlm.nih.gov/sra/?term=SRR5188379) and the simulated data from the mock metagenome dataset is available from the University of Maryland Institute for Genome Sciences in Dryad doi:10.5061/dryad.m1m0p. Tables S1, S2, and S3 are available in the online Supplementary Material.

## Impact Statement

As most organisms do not live in isolation, it can be informative to study combinations of organisms in co-culture. This has been greatly facilitated by ever improving sequencing data and tools. However, many of these tools were not developed for multi-species datasets, and it is not immediately apparent that optimization is needed or what optimization is needed. How multi-species datasets are handled varies even between generations of the same aligner, like the Burrows–Wheeler aligner (bwa)- aln and bwa- mem (maximum exact matches). In this case, optimization could be obtained with bwa- mem by using a combined reference, containing all of the genomes of the species present, and changing the seed stringency. There are many uses for aligners for multi-species datasets that will benefit from this optimization including: (a) use in dual-species RNASeq datasets where mis-mapping leads to inappropriate gene expression calculations, (b) use in identifying discordant read pairs that suggest horizontal/lateral gene transfer, and (c) use to cull datasets, for instance removing human reads from clinical pathogen data sets prior to data deposition. All of these cases will benefit from the increased specificity of the optimization here with little loss to sensitivity.

## Introduction

One of the most popular tools for aligning genome sequencing reads to a reference genome is the Burrows–Wheeler aligner (bwa). The bwa software package allows users to align highly similar sequences to a large reference genome based on searches for exact matches with the Burrows–Wheeler transform [1]. There are three different bwa algorithms: bwa-aln (or bwa-backtrack), bwa-sw (Smith–Waterman Alignment) and bwa-mem (maximum exact matches). bwa-aln was the original bwa algorithm and was developed for 36–100 bp reads [1]. bwa-aln allows for gaps and mismatches, facilitating the identification of insertions and deletions [1]. When originally implemented, bwa-aln was 10–20× faster than the leading alignment tool, without sacrificing accuracy [1].

bwa-sw was originally used for mapping longer reads, like those originating from the 454 platform, but was updated for use with 200–1 000 000 bp sequencing reads [1]. As the length of sequencing reads increases, so does the error rate and the potential for structural variation within the reads. Therefore, alignment algorithms for longer reads must switch from global matches to local matches to accommodate this error rate and potential structural variation [1]. bwa also implemented a Smith–Waterman-like dynamic program to quickly align the query sequence to all suffix trees of the reference [1]. In addition to this seed-and-extend approach, heuristics were added to further increase the speed of bwa-sw [1]. However, as the amount of data generated by large-scale genomics projects increased and read length decreased, bwa-sw lacked the ability to analyze the data [1].

The most recent addition to the bwa suite is bwa-mem [2]. bwa-mem was implemented to increase the speed of aligning 100–1000 bp sequences to a large reference genome, while maintaining accuracy [2]. bwa-mem has the ability to perform chimeric alignments, can handle sequencing errors, and can choose between local or global/end-to-end alignments [2]. It is ideal for sequencing reads >70 bp and can handle reads up to 1 Mbp [2]. bwa-mem uses a seed-and-extend approach and extends the seed with an affine-gap Smith–Waterman algorithm, but also implements re-seeding to reduce incorrect mappings from a missing seed [2]. bwa-mem also employs heuristics that prevent it from extending an alignment through a poorly mapping area [2]. Another significant improvement of bwa-mem is that larger reference databases can be used. With bwa-aln, the complete reference index was limited to around the length of the human genome [1], but bwa-mem can now handle longer references [2]. bwa-mem is reported to be much faster than other aligners, and provides accurate alignments for both short and long reads [2].

Due to the speed and accuracy of bwa, it has been the aligner of choice for many different types of analyses from various organisms. bwa has been used for the analysis of all phases of the 1000 Genomes Project, studying multiple types of genetic variation in thousands of individuals across the globe [3–5]. In addition to studying this genetic variation, bwa has also been used to align cancer genome sequences to the human genome, including ovarian cancer [6], stomach cancer [7], prostate cancer [8], renal-cell cancers [9–11], cutaneous melanoma [12], head and neck squamous cell carcinoma [13], lung cancers [14, 15], papillary thyroid cancer [16], urothelial bladder carcinoma [17], acute myeloid leukemia [18], colon and rectal cancer [19], breast cancer [20], endometrial cancer [21], and glioblastoma [22].

While bwa was initially designed for single nucleotide polymorphism (SNP)-based analysis [2], it has also been used to identify structural variation. The Mobile Element Locator Tool (melt) [5, 23], which identifies mobile elements in eukaryotic sequencing samples, relies on bwa-generated alignments. melt was used in the 1000 Genomes Project to identify structural variants and LINE element insertion and deletion events in these samples [5, 23]. LGTSeek relies on bwa-aln to align read pairs to identify lateral gene transfers (LGTs) from a donor organism to a recipient organism [24]. LGTSeek can align sequencing reads to the recipient reference, then align the unmapped reads to the potential donor references, identifying pairs where one read maps to the host and the other read maps to the donor [24]. This approach relies on the speed and accuracy of bwa-aln to accurately identify these categories of reads.

While bwa-mem aligns the sequencing reads to the reference more quickly than bwa-aln [1], we have observed instances of low stringency/specificity of these alignments when using LGTSeek. This decreased specificity can result in the double mapping of sequencing reads to multiple references. For example, this was observed while examining dual-species transcriptome data [25] from *Wolbachia* endosymbionts and *Drosophila ananassae*, and was solved by combining the reference genomes. In this case, in preliminary work, bwa-aln and bwa-mem were both used for the alignments, but the counts were wildly different. More specifically, bwa-mem mapped bona fide *Wolbachia* sequencing reads to the *D. ananassae* reference genome, when only a very small portion of the read matched the *Drosophila* reference genome. This likely results from the fact that bwa was designed with the intent of mapping sequence data from a single organism to a single reference genome. However, as more tools use bwa for multi-species comparisons and more researchers use it in host–pathogen samples, these misalignments can significantly confound analyses.

Here, we sought to optimize bwa-mem in eukaryote/eukaryote and eukaryote/prokaryote dual-species datasets by altering various options to prevent incorrect mappings. We identified appreciable effects upon altering the seed length of bwa-mem alignments, which determines the minimum base pair length of an exact match, and by default is 19 nt. Since bwa-mem can handle larger reference databases, we also separately analyzed the effect of combining host–pathogen references into one larger database. Both methods decrease computational time, while increasing or maintaining specificity of the alignments. During this research, we also observed that the computational time spent on human genome sequence alignments, in general, could be decreased by increasing the seed length, which we attribute to the time spent attempting to align poor quality, off-target, non-human contaminant sequences to the reference genome.

## Methods

### Sample description

#### Human dataset

The 100 bp paired-end Illumina human dataset is available from the 1000 Genomes Project in the Sequence Read Archive (SRA) (http://www.internationalgenome.org, ERR0 22446). The data is from a Colombian male and the sample (HG01133) of B-lymphocytes was collected from the peripheral vein [5].

#### Plasmodium-human dataset

The 76 bp paired-end Illumina *Plasmodium–*human datasets are available from the Wellcome Trust Sanger Institute in the SRA (ERR015379, ERR012739 and ERR015360) from a study that extracted DNA from leukocyte-depleted blood samples or after short-term *in vitro* culture [26]. These samples (PK0055-C, PK0076-C and PK0044-C) all originated in Burkina Faso in 2008 [26].

#### *Brugia*– *Wolbachia* dataset

The 101 bp paired-end *Brugia–Wolbachia* dataset is available from the University of Maryland Institute for Genome Sciences in the SRA (SRR5188379) from a currently unpublished study designed to test DNA amplification methods on low input DNA samples. DNA was extracted from a population of *Brugia malayi* worms. Qiagen Repli-G (Qiagen) was used to amplify 2 ng genomic DNA with tetramethylammonium chloride (TMAC)[27]. A genomic DNA library was constructed for sequencing on the Illumina platform using the KAPA Hyper Prep kit (Kapa Biosystems) according to the manufacturer’s protocol. DNA was fragmented with the Covaris E210. The DNA was purified between enzymatic reactions and the size selection of the library was performed with SPRI-select beads (Beckman Coulter Genomics). The PCR amplification step was performed with primers containing a 7 nt index sequence. The indexed libraries were pooled and sequenced on a on a Hiseq2500 sequencer (Illumina).

#### Simulated metagenomic dataset

A dataset of 101 bp paired-end sequencing reads from a mock metagenomic community was simulated with art, version 2.3.7 28]. An even 8× sequencing depth with a HiSeq2500 error profile, a minimum fragment length of 300 bp and sd of 105 bp was generated across the reference genomes: *Acinetobacter baumanii* ATCC17978; *Actinomyces odontolyticus* ATCC17982; *Bacillus cereus* ATCC10987; *Bacteroides vulgatus* ATCC8482; *Clostridium beijerinckii* ATCC51743; *Deinoccous radiodurans* DSM20539*; Enterococcus faecalis* ATCC47077; *Escherichia coli* ATCC700926; *Helicobacter pylori* ATCC700392; *Lactobacillus gasseri* DSM 20243; *Listeria monocytogenes* ATCCBAA-679; *Methanobrevibacter smithii* ATCC35061; *Neisseria meningitidis* ATCCBAA-335; *Propionibacterium acnes* DSM16379; *Pseudomonas aeruginosa* ATCC47085; *Rhodobacter sphaeroides* ATCC17023; *Staphylococcus aureus* ATCCBAA-1718; *Staphylocococcus epidermidis* ATCC12228; *Streptococcus agalactiae* ATCCBAA-611; *Streptococcus mutans* ATCC7 00610; and *Streptococcus pneumoniae* ATCCBAA-334.

### bwa alignments

All alignments were written as sam output files using bwa v. 0.7.12. The qsub -m function, as implemented in GE 6.2u5 lx24-amd64 dated December 1, 2009, was used to generate a summary report from each experiment including the central processing unit (CPU) time for each script that was executed. To reduce variation in CPU times, all jobs were executed on a single multi-core node ensuring that <40 % of the total number of cores and <75 % of total memory were used. Each job was executed in triplicate, and no replicate jobs were running simultaneously. For bwa-aln/sampe alignments, the script contained aln commands for each fastq file executed sequentially followed by the sampe command, which were all executed with the default parameters. For bwa-mem alignments, the script contained a single command to align the sequencing read pairs to their respective references; default parameters were used except where seed length was altered. The Ensembl GRCh37 human reference containing the decoy sequence was used for alignments to the human genome. The *B. malayi* genome [29] version 4 was obtained from WormBase (www.wormbase.org) and was used as the combined reference for *Brugia* and *Wolbachia*. The *Wolbachia w*Bm genome [30] reference was obtained by pulling the Bm_006 contig from the combined reference and the *Brugia* reference was made by using all contigs except Bm_006. The *Plasmodium* reference used was version 3.0 of high quality *Plasmodium falciparum* 3D7 draft genome released by PlasmoDB-9.3 [31]. The combined human–*Plasmodium* reference was created by adding the *Plasmodium* contigs to the GRCh37 fasta file. samtools flagstat v.1.1 was used to determine the number of total reads, secondary reads, and mapped reads in each sample. To correct for the number of secondary reads, the percentage of reads mapping was calculated as (mapped reads – secondary reads)/(paired in sequencing reads) ×100.

## Results

### Datasets

Four types of datasets were used for this study: eukaryote/prokaryote, eukaryote/eukaryote, eukaryote only and simulated metagenomic. The first dataset contained 101 bp paired-end Illumina sequencing reads from a population of *B. malayi* worms and their *Wolbachia* endosymbiont strain *w*Bm from an unpublished study examining DNA amplification methods; it will be referred to as the *Brugia–* *Wolbachia* dataset (Table 1). Using the Perl 5.8.8 rand() and srand() functions, 250 000 first reads were randomly selected and the taxonomy of best hits for these reads was generated with a mega-blast (v. 2.2.17) search against the NCBI nucleotide database NT. We typically find that in mega-blast searches of this nature that some percentage of reads have no match, and that the best criterion to use is the percentage of reads with a best match relative to the number of reads that have any match, and this criterion was used in all other instances. Based on this criterion, this dataset was estimated to contain 79.7 % reads with matches having best matches to *B. malayi* and 14.5 % reads with best matches to *Wolbachia w*Bm. However, the version of NT used for these searches does not have the *B. malayi* genome, so most reads did not have matches, making this measurement skewed in this case. If we instead focus on all reads, 14.1 % of reads had a best match to *B. malayi* and 2.6 % of reads had a best match to *Wolbachia w*Bm, which is likely a better reflection of the composition of this data.

**Table 1.** Total number of reads for each dataset The datasets are listed that were used in this study, with the total number of reads.

**Dataset**	**Accession no.**	**Total no. of reads**
*Brugia–Wolbachia*	SRR5188379	31 494 916
*Plasmodium–*human	ERR015379	6 051 406
*Plasmodium–*human	ERR012739	8 080 550
*Plasmodium–*human	ERR015360	25 867 194
1000 Genomes	ERR022446	219 493 146
Simulated metagenome	Dryad acc. (doi:10.5061/dryad.m1m0p)	5 490 726

The second dataset that we initially focused on contained 76 bp paired-end Illumina sequence reads from *P. falciparum* sequenced from a human sample from the SRA (ERR015379) [26]; it will be referred to as the *Plasmodium–*human dataset (Table 1). Parsing the taxonomy of best hits of 250 000 randomly selected first reads generated with a mega-blast search against NT, this dataset was estimated to contain 87.5 % reads with best matches to *P. falciparum* reads and 7.4 % reads with best matches to human sequences.

The third dataset contained 101 bp paired-end Illumina sequence reads that have been simulated from a mock metagenomic community (Dryad doi:10.5061/dryad.m1m0p); it will be referred to as the mock metagenomic dataset (Table 1). The final dataset examined was 100 bp Illumina paired-end human sequencing reads from the 1000 Genomes Project (ERR022446) [5]; it will be referred to as the 1000 Genomes dataset (Table 1). Parsing the taxonomy of best hits of 250 000 randomly selected first reads generated with a mega-blast search against NT, this dataset was estimated to contain 94.7 % of reads with matches having best matches to human sequences.

### Percentage of mapped read pairs using different seed lengths with bwa-mem

**Fig. 1.**
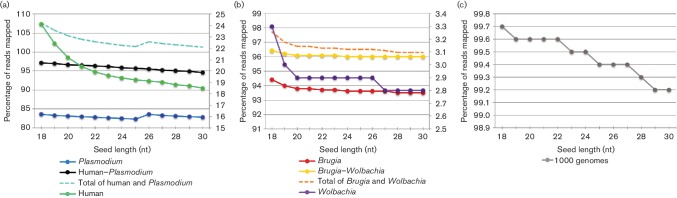
The percentage of mapped read pairs for all bwa-mem datasets. The percentage of mapped read pairs for all datasets against all references is shown for each seed length for (a) the human–*Plasmodium* dataset, (b) the *Brugia–Wolbachia* dataset and (c) the human-only dataset. The sequencing reads were aligned to the reference genome of each species separately, as well as a combined reference of both genomes, as indicated in the legend. Mappings to the reference genome of the minority member in the sample (human for the *Plasmodium–*human dataset and *Wolbachia* for the *Brugia–Wolbachia* dataset) were plotted on the secondary *y*-axis on the right, while all others were plotted on the primary *y*-axis on the left. The sum of the mappings to individual reference genomes is illustrated with a dashed line to enable comparisons.

bwa-mem was used to align three different whole genome sequencing datasets to the respective reference databases with varying seed lengths from 18 to 30 nt. In the two dual-species datasets, the sequencing reads were aligned to the reference genome of each species separately, as well as a combined reference of both genomes. The 1000 Genomes dataset was only aligned to the human reference genome. The lowest variation in percentage of reads mapped with bwa-mem was observed in the 1000 Genomes dataset, where the total percentage of reads mapped only varied by 0.49 % across all seed lengths tested (Fig. 1, Table 2).

**Table 2.** The percentage of read pairs that aligned to each reference The percentage is provided of read pairs from a subset of datasets that aligned to each reference for bwa-aln with default parameters and bwa-mem with seed lengths from 18 to 30 nt.

**Dataset**	**Reference**	**aln**	**18**	**19**	**20**	**21**	**22**	**23**	**24**	**25**	**26**	**27**	**28**	**29**	**30**
*Plasmodium–*human	Human	10.2	24.1	22.4	21.2	20.4	19.9	19.6	19.4	19.2	19.1	19.0	18.8	18.7	18.5
*Plasmodium–*human	*Plasmodium*	78.1	83.6	83.3	83.1	82.9	82.8	82.6	82.4	82.3	83.6	83.3	83.1	82.9	82.8
*Plasmodium–*human	Combined reference	88.3	97.1	96.9	96.7	96.5	96.3	96.1	95.9	95.7	95.5	95.3	95.1	94.9	94.6
*Brugia–Wolbachia*	*Brugia*	91.9	94.4	94.0	93.8	93.8	93.7	93.7	93.6	93.6	93.6	93.6	93.5	93.5	93.5
*Brugia–Wolbachia*	*Wolbachia*	2.7	3.3	3.0	2.9	2.9	2.9	2.9	2.9	2.9	2.9	2.8	2.8	2.8	2.8
*Brugia–Wolbachia*	Combined reference	94.5	96.4	96.2	96.1	96.1	96.1	96.1	96.0	96.0	96.0	96.0	96.0	96.0	96.0
1000 Genomes	Human	95.9	99.7	99.6	99.6	99.6	99.6	99.5	99.5	99.4	99.4	99.4	99.3	99.2	99.2

In the *Brugia–* *Wolbachia* dataset, reads mapping to the reference genome of the minority member (*Wolbachia*) decreased markedly from 3.3 to 2.9 % when increasing the seed length from 18 to 20 nt (Fig. 1, Table 2). While this is only a 0.4 % difference, it is important to consider it is >10 % of the reads that mapped to this genome. This problem of read pairs aligning to both references was resolved, meaning they were mapped to the best position, by combining the *Brugia* and *Wolbachia* references into a single reference sequence (Fig. 1, Table 2). The percentage of reads that mapped to *Brugia* only varied by 0.87 % across all seed lengths (Fig. 1, Table 2). However, the extensive amount of recent LGT to *B. malayi* from its *Wolbachia* endosymbiont [32] confounds this analysis such that we sought to evaluate this phenomenon with several subsequent datasets.

In the *Plasmodium–*human dataset, the aggregate number of read pairs aligning to each reference was >100 % until the seed length was set to >29 nt, when mapping drastically declined due to a likely over-stringent seed length. The aggregate number of read pairs decreased from 107.8 to 102.2 % when the seed length was increased from 18 to 23 nt (Fig. 1, Table 2). This problem of read pairs aligning to both references was resolved by combining the human and *Plasmodium* reference into one file (Fig. 1, Table 2). For all seed lengths mapped against this combined reference, the *Plasmodium–*human dataset consistently had 94.6–97.1 % of read pairs aligning (Fig. 1, Table 2). Aligning the *Plasmodium–*human data to the *Plasmodium* reference or the *Plasmodium–*human combined reference led to a consistent percentage of read pairs mapping across seed length up to 30 nt, indicating that increasing the seed length did not dramatically decrease the number of read pairs that were aligned for these seed lengths (Fig. 1, Table 2). Increasing the seed length beyond 30 nt led to a substantial decrease in the number of reads aligned, as expected (Table S1, available in the online Supplementary Material), which was only tested with this dataset.

**Fig. 2.**
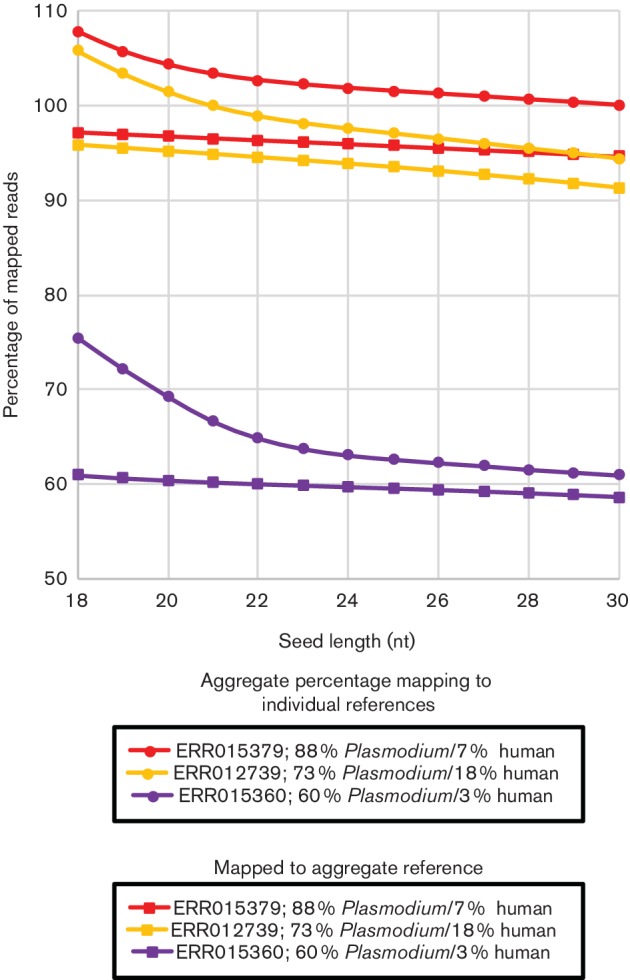
Percentage of mapped read pairs for different *Plasmodium–*human datasets. The percentage of mapped read pairs for all datasets against all references is shown for each seed length for three *Plasmodium* datasets containing varying levels of human sequence. ERR015379 is estimated to contain 88 % reads with best matches to *P. falciparum* reads and 7 % reads with best matches to human sequences. ERR012739 was estimated to contain 73 % *Plasmodium* reads and 18 % human reads. ERR015360 was estimated to contain 60 % *Plasmodium* reads and 3 % human reads. All three datasets showed the same trends as the original dataset examined, namely over-mapping of reads when mapped to separate references that could be largely eliminated by mapping to an aggregate reference and/or increasing the seed length to 23 nt.

Two further *Plasmodium–*human datasets were tested, ERR012739 and ERR015360, to ensure these observations were not limited to the original dataset selected. Parsing the taxonomy of best hits of 250 000 randomly selected first reads generated with a mega-blast search against NT, ERR012739 was estimated to contain 73 % *Plasmodium* reads and 18 % human reads, while ERR015360 was estimated to contain 60 % *Plasmodium* reads and 3 % human reads. All three datasets showed the same trends as the original dataset examined (Fig. 2), namely over-mapping of reads when mapped to separate references that could be largely eliminated by mapping to an aggregate reference and/or increasing the seed length to 23 nt.

**Fig. 3.**
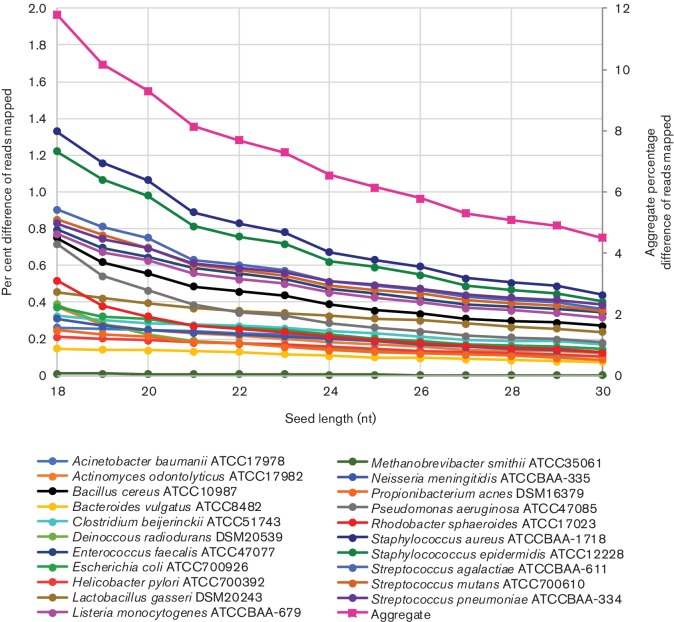
The difference in the percentage of mapped reads from that expected for simulated reads from a mock metagenomic community. Because of differences in the genome size, the percentage of reads mapped varied extensively, such that the difference between the observed percentage of mapped read pairs and the known percentage of mapped read pairs was interrogated for the simulated data from the mock metagenomic community. This value is shown for each seed length for a simulated dataset of 101 bp paired-end sequencing reads from a mock metagenomic community. Given that the data is simulated, the known percentage of mapped reads was calculated from the 8× sequencing depth and the relative fraction the genome contributes to the population for each organism. The aggregate percentage difference in mapping was plotted on the secondary *y*-axis on the right, while all individual percentage differences were plotted on the primary *y*-axis on the left.

Similar over-mapping of reads was again observed with a simulated dataset of a 21-member mock metagenomic community (Fig. 3). When mapped to the composite reference, 100 % of the reads mapped, as expected for a simulated dataset. However, even at a seed length of 30 nt, the aggregate percentage of reads mapping to each individual reference was still >104 % (Fig. 3), which may be due in part to multi-mapping of reads to the many highly similar rRNAs. Mapping to an aggregate reference and/or increasing the seed length to 23 nt improved the mapping specificity (Fig. 3). However, it is less clear that 23 nt was the ideal seed length as substantial improvements could be observed beyond a 23 nt seed length (Fig. 3). This difference may be due to using simulated sequence data, the high degree of similarity in regions of the genomes like the rRNA, or the complexity of the population. We investigated using real sequencing data from a mock community sequenced by the human microbiome project [33, 34], but only single end reads were available.

### CPU times and trends

**Fig. 4.**
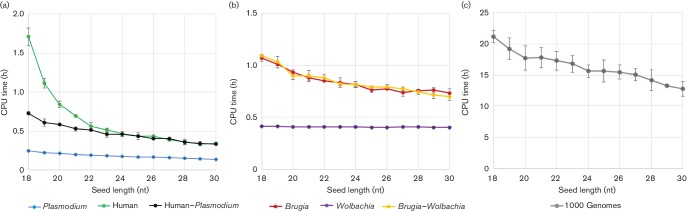
CPU time for all bwa-mem datasets. The mean and sd of CPU time in hours for each of three replicate alignments is plotted against each seed length for a subset of datasets. In the two dual-species data sets, the sequencing reads were aligned to the reference genome of each species separately, as well as a combined reference of both genomes. The 1000 Genomes dataset was only aligned to the human reference genome.

The CPU time of the alignments was also interrogated, by measuring in triplicate using a single grid node with 48 CPUs and 95.6 GB memory, controlling for variation by limiting CPU usage to <40 % of the maximum and memory usage to <75 % of the maximum (Fig. 4). We could not control for other factors like caching and network traffic, but saw little variation in CPU time across replicates, which were not run simultaneously on the node. The alignment of the reads to the combined *Brugia–Wolbachia* reference database took similar CPU time relative to aligning to only the *Brugia* reference (Fig. 4, Table 3) demonstrating the benefit of using a combined reference when possible.

**Table 3.** The CPU time in hours for each replicate for a subset of datasets and references The CPU time in hours is provided for a subset of datasets for bwa-aln with default parameters and bwa-mem with 18–30 nt seed lengths. For bwa-aln, the CPU time reported is the sum of the CPU time for aligning read 1, the CPU time for aligning read 2 and the CPU time for running the sampe algorithm on those two alignments.

	**aln**	**18**	**19**	**20**	**21**	**22**	**23**	**24**	**25**	**26**	**27**	**28**	**29**	**30**
*Plasmodium*	0.3±0.1	0.2±0	0.2±0	0.2±0	0.2±0	0.2±0	0.2±0	0.2±0	0.2±0	0.2±0	0.2±0	0.1±0	0.1±0	0.1±0
Human	0.9±0.1	1.7±0.1	1.1±0.1	0.8±0	0.7±0	0.6±0	0.5±0	0.5±0	0.4±0	0.4±0	0.4±0	0.4±0	0.3±0	0.3±0
Human–*Plasmodium*	1±0.1	0.7±0	0.6±0	0.6±0	0.5±0	0.5±0	0.5±0	0.5±0	0.4±0	0.4±0	0.4±0	0.4±0	0.3±0	0.3±0
1000 Genomes	34.1±1	21.1±1.7	19.2±1.9	17.7±1.6	17.8±1.5	17.3±1.4	16.8±1	15.6±1.8	15.7±1.1	15.5±1	15.1±1.6	14.2±0.1	13.3±1.2	12.8±1.1
*Brugia*	1.3±0	1.1±0	1±0	0.9±0	0.9±0	0.9±0	0.8±0	0.8±0	0.8±0	0.8±0	0.7±0	0.8±0	0.8±0	0.7±0
*Wolbachia*	0.1±0	0.4±0	0.4±0	0.4±0	0.4±0	0.4±0	0.4±0	0.4±0	0.4±0	0.4±0	0.4±0	0.4±0	0.4±0	0.4±0
*Brugia–* *Wolbachia*	1.3±0	1.1±0.1	1±0	0.9±0.1	0.9±0	0.9±0	0.8±0	0.8±0	0.8±0	0.8±0	0.8±0	0.7±0	0.7±0	0.7±0

Alignments to the human reference genome had the most dramatic decrease in CPU time with increasing seed lengths for both the human–*Plasmodium* dataset and the 1000 Genomes dataset (Fig. 4). In the case of the 1000 Genomes dataset, the CPU time at a seed length of 18 nt was 21.1±1.7 h (Table 3). Increasing the seed length to 23 nt reduced the CPU time to 16.8±1.0 h, resulting in a 1.3-fold reduction or 4.3 h decrease in CPU time (Fig. 4, Table 3).

The *Plasmodium–*human dataset showed even more substantial improvements when aligning to the combined dataset, but most substantially between 18 and 20 nt seed lengths. For example, at a seed length of 18 nt, the alignment of this dataset to the human reference took 1.7±0.1 h, while the alignment to the combined reference only took 0.7±0 h, a 2.4-fold decrease (Table 3, Fig. 4). The seed length also plays a role as observed by the decrease in CPU hours with increasing seed length up to ~23 nt. An increase in seed length to 23 nt reduced the *Plasmodium–*human CPU time 3.4-fold in the alignments to just the human reference (Fig. 4, Table 3). Overall, improvements to the CPU time for both host*–*pathogen datasets plateaued by seed length of 23 nt (Fig. 4, Table 3). The decreasing CPU time needed as a function of seed length, combined with the over-mapping of reads against a single reference compared to a combined reference, suggests that using bwa-mem to generate poor alignments to the incorrect reference can lead to excessive use of computational resources. Increasing the seed length beyond 30 nt led to a sizable decrease in the CPU hours (Table S2), but this is likely a result of the undesirable decrease in the number of reads aligned (Table S1).

### Comparing bwa-mem to bwa-aln

We also sought to compare the percentage of mapped read pairs aligned with bwa-aln and bwa-mem. We expected that bwa-mem would map more reads, since it could map reads with more sequencing errors, as well as greater sequence variation. Consistent with these expectations, in all cases tested, bwa-aln mapped a lower percentage of reads relative to bwa-mem (Table 2). Also, as expected, given the underlying algorithm, bwa-mem computed the alignments faster than bwa-aln when the majority of reads map to the reference genome (i.e. *Plasmodium–*human against the *Plasmodium* genome, *Plasmodium–*human against the combined reference, *Brugia–Wolbachia* against the *Brugia* genome, *Brugia–Wolbachia* against the combined reference, and 1000 Genomes against the human genome). Conversely, we anticipated that when the vast majority of reads did not map to the reference, bwa-mem would spend large amounts of compute time trying to incorrectly align them to the genome (i.e. *Plasmodium–*human against the human genome and *Brugia–* *Wolbachia* against the *Wolbachia* genome). As expected, these latter two cases were aligned more quickly with bwa-aln than bwa-mem at the lower seed lengths tested (Table 3).

The increase in the percentage of reads mapping with bwa-mem at a seed length of 18 nt led to an increase in both the sequencing read depths, as well as the coverage. There are 12 127 496 positions in the human genome that had 1× sequencing depth with a seed length of 18 nt using bwa-mem with a maximum sequencing depth of 7990× with the default parameters of samtools mpileup, which would discard positions with >8000× sequencing depth. In contrast, 5 378 722 positions in the human genome had 1× sequencing depth with bwa-aln/sampe with a maximum sequencing depth 919×. Therefore, there was >8-fold higher maximum sequencing depth and >2-fold more positions with coverage.

The *Plasmodium* reads mapping to the human genome with a seed length of 18 nt were not evenly distributed throughout the human genome. While reads mapped to all of the chromosomes evenly, three non-chromosomal fasta entries had reads that were significantly over-represented relative to the length of the sequence (χ^2^, *P*<0.00005), including the unplaced genomic contig GL000220.1 with 69 056 reads in 161 802 bp, the unplaced genomic contig GL000226.1 with 16 735 reads in 15 008 bp, and the decoy sequence contig hs37d5 with 569 969 reads in 35 477 943 bp.

We further wanted to examine whether high-quality matches of *Plasmodium* reads to the human genome reference enabled alignment of related poorly mapping reads through any of bwa’s processes like chaining, filtering chaining, and seed extension. We used the 10.2 % of *Plasmodium* reads aligning to the human reference with bwa-aln as our proxy for high-quality matches. Those reads and their mates were removed from the fastq files and these modified human*–* *Plasmodium* fastq files were realigned to the human genome using bwa-mem for all seed lengths tested. We found that the percentage of reads mapping to the human reference with bwa-mem was the same from both the filtered fastq files and the initial fastq files after accounting for the removal of the 10.2 % of the reads that correctly aligned to *Plasmodium* (Table S3).

There was no substantial difference between mapping to the *Plasmodium* genome with bwa-aln/sampe and bwa-mem. There were 16 995 093 *Plasmodium* positions with >5× coverage in the bwa-mem alignment with a seed length of 18 nt, while there were 16 698 169 *Plasmodium* positions with >5× coverage in the bwa- aln/sampe alignment. When modified fastq files were made by removing reads identified as human using bwa-aln/sampe and those modified files were mapped with bwa-aln/sampe back to the *Plasmodium* genome, 16 698 614 *Plasmodium* positions still had >5× coverage, which is remarkably similar to the 16 698 169 *Plasmodium* positions with >5× coverage when mapping all reads to the *Plasmodium* genome without filtering. However, when modified fastq files were made after removing reads identified as human using bwa-mem and those modified files were mapped with bwa-aln/sampe back to the *Plasmodium* genome, only 14 214 800 *Plasmodium* positions still had >5× coverage, which is only 85 % of the positions with coverage in the *Plasmodium* genome using all reads and bwa- aln/sampe. Therefore, the excess removal of reads based on their alignment with bwa-mem at a seed length of 18 nt may result in a loss of coverage of ~2 000 000 *Plasmodium* positions that could lead to an inability to call SNPs and other important genetic variants.

## Discussion

### Effects on multi-species genome sequencing datasets

New algorithms are making remarkable advances to expand the complexity of alignments to best capture all the genomic variation in a single organism [1, 2, 35–38]. These improved alignments are essential for better identification of SNPs, copy number variants and instances of structural variation. However, there is the potential for problems to arise when these algorithms are applied in untested scenarios, like multi-species sequencing projects. In these cases, one of the vital steps is properly attributing the reads to each organism.

Numerous studies have reported transcriptomics-based gene expression differences of two organisms within a single sample [39–50]. For the gene expression differences to be accurately obtained, the reads from both organisms need to map uniquely to the appropriate reference. The results presented here for *Brugia–* *Wolbachia* and *Plasmodium–*human read mappings suggests this may not occur all the time. *Plasmodium* reads map to the human genome and *Brugia* reads map to the *Wolbachia* genome, which could result in transcripts appearing to be transcribed that are not. It also could result in erroneous assessment of the level of differential gene expression.

This may also present problems in mapping-based metagenomics analyses, and our results suggest that using a combined reference and increasing the seed length may lead to improvements. The exact seed length is not clear, and further work using real sequencing data from mock communities, as opposed to simulated data, may be necessary to resolve the matter.

In parasite and pathogen sequencing projects that require the direct isolation of nucleic acids from a human patient, sequencing reads are often screened prior to deposition in public repositories, like the SRA [51], to remove human sequences. Theoretically, mapping-based algorithms, like bwa, provide fast and efficient mechanisms for removing such human reads from parasite and pathogen sequencing projects. However, the results presented here suggest that too many sequences may be erroneously targeted for removal. Specifically, only 85 % of the positions have coverage in the *Plasmodium* genome after removing ‘human’ reads with bwa-mem with a seed length of 18 nt relative to removing ‘human’ reads with bwa- aln/sampe.

Lastly, pipelines like PathSeq [52] and LGTSeek [24] use aligners to identify the putative provenance of reads, assigning a taxonomy for reads and/or read pairs. PathSeq identifies microbial reads in samples, in order to identify novel microbe–disease associations [52]. LGTSeek identifies read pairs with discordant taxonomy, like bacteria-eukaryote read pairs in animal and human genomes that may indicate bacteria-animal LGT[24]. In this case, a stringent mapping assignment is needed in order to assign the most appropriate taxonomy. The results from both the *Brugia–* *Wolbachia* and the *Plasmodium–*human datasets suggest that this may not be the case with bwa-mem without further optimization. While we focus on differences between bwa-aln/sampe and bwa-mem, it is important that any aligner be thoroughly tested when used in these scenarios for the particular organisms under study.

### Solutions

Given the results presented here, one solution that should be employed whenever possible is to include the genomes of all expected organisms in a single reference database. Doing so increased the specificity of the alignments and decreased computation times. This strategy has already been used in human genome sequencing projects that include the Broad Institute’s ‘decoy’ genome sequence in their reference genome. The ‘decoy’ genome sequence contains sequences frequently encountered in human whole-genome sequencing projects that are otherwise not in the reference [5].

In some studies, however, not all reference organisms are known. For example, when running algorithms like PathSeq [52] or LGTSeek [24] to identify instances of ‘foreign’ DNA, the potential sources are unknown and are the focus of the study. Additionally, if a sequencing library or run is contaminated, the researcher may not be aware of the presence of sequence reads from additional organisms that could be leading to an increase in computation times. In these cases, the specificity would be improved and the CPU times decreased by increasing the seed length. In this study, a seed length of 23 nt was ideal since lower seed lengths increased computation times and off-target mappings, while higher seed lengths could reduce sensitivity with no improvement to computation times or off-target mappings. It is not clear if this is the universal ideal seed length that would be applicable for all scenarios. The ideal seed length may be a function of the evolutionary distances and similarity between the organisms being sequenced concomitantly. However, we have demonstrated that the use of simulated datasets constructed from the reference datasets may be useful in informing these decisions and identifying what further experimental controls may be needed.

While much of our analysis focuses on a seed length of 18 nt, it is important to remember that the default seed length for bwa-mem is 19 nt. We initially identified this problem with a seed length of 19 nt; however, it is much more apparent and easier to interrogate at a seed length of 18 nt. Lastly, there may be scenarios that warrant the use of legacy aligners, like bwa- aln, which may be less sensitive, but have a higher degree of specificity.

### Computational resource optimization

Analysis of genomic data can be computationally intensive, leading to long CPU times and high analysis costs. Our analysis suggests that almost all datasets can benefit from an increase in the seed length to reduce CPU time and associated costs. However, this decrease in computation time may affect the ability to identify ‘split reads’, where a read matches two different locations in the genome, suggesting structural variation in genomics projects and splicing in transcriptomics projects. As such, further work may be needed by specialists in a field (e.g. structural genomic scientists and annotation specialists) to demonstrate how changing the seed length may or may not affect the validity of structural variation and gene structure predictions. Split reads in RNASeq projects of dual-species projects need to be handled with care, balancing the sensitivity of detecting splice junctions with the specificity of correct mapping. At a minimum, this can be dealt with in multi-species datasets by aligning to a combined reference that contains all known members of the consortium sequenced.

### Conclusion

As sequencing technologies continue to advance and sequencing becomes less expensive, the tools to analyze these sequences need to be optimized for the intended study. Here, bwa-mem was optimized to reduce alignment time and increase the specificity of alignments. While this optimization might not be necessary for all datasets, we found it to be particularly helpful in sequencing data obtained simultaneously from multiple organisms. In these cases, there was one dominant sequence source (i.e. *Plasmodium* or *Brugia*) and one minority sequence source (i.e. human or *Wolbachia*). Increasing the seed length reduced the number of read pairs aligning, incorrectly, to the reference genome from the minority sequence source (human or *Wolbachia*). Overall, increasing the seed length to 23 nt and combining both organisms into one reference genome can reduce the CPU time, sometimes quite substantially, with more specificity in the mapping, as was the case with the *Plasmodium–*human dataset. In some situations, the use of legacy aligners may also be warranted. Less substantial, but still possibly significant, improvements to CPU time upon changing the seed length were found in all cases. This suggests using a 23 nt seed length by default may be desirable.

## Data bibliography

1. 1000 Genomes Project. Wellcome Trust Sanger Institute. Sequence Read Archive – ERR022446 (2017).
2. Wellcome Trust Sanger Institute. Sequence Read Archive – ERR015379 (2017).
3. Wellcome Trust Sanger Institute. Sequence Read Archive – ERR012739 (2017).
4. Wellcome Trust Sanger Institute. Sequence Read Archive – ERR015360 (2017).
5. University of Maryland Institute for Genome Sciences. Sequence Read Archive – SRR5188379 (2017).
6. University of Maryland Institute for Genome Sciences. Dryad – doi:10.5061/dryad.m1m0p (2017).

## Supplementary Data

273 Supplementary File 1
