# Supplementary material for: Aligner optimization increases accuracy and decreases compute times in multi-species sequence data
Source: Microb Genom. 2017 Jul 8;3(9):e000122. doi: 10.1099/mgen.0.000122 (PMC5643015; doi:10.1099/mgen.0.000122)
Supplement: Supplementary File 1 [file mgen-3-122-s001.pdf]

Supplementary Table 1. The Percentage of Read Pairs for Seed Lengths >30 Nucleotides.

The percentage is provided of read pairs for one of the *Plasmodium*-human datasets (ERR015379) aligned to each reference for BWA-ALN with default parameters and BWA-MEM with seed lengths from 18-30 nucleotides.

**Reference**	**40**	**50**	**60**	**70**	**72**	**74**	**75**	**76**
Human	13.1	10.2	7.9	5.0	4.7	4.5	4.4	4.3
Plasmodium	90.8	85.6	79.4	66.1	64.8	63.6	63.0	62.2
Combined Reference	79.0	75.7	71.5	61.1	60.1	59.1	58.6	58.0

Supplementary Table 2. The CPU Time in Hours for Seed Lengths >30 nucleotides.

The CPU time in hours is provided for one of the *Plasmodium*-human dataset (ERR015379) and references for BWA-MEM with seed lengths >30 nucleotides.

**Reference**	**30**	**40**	**50**	**60**	**70**	**72**	**74**	**75**	**76**
Plasmodium	0.1 ± 0	0.1 ± 0	0.1 ± 0	0.1 ± 0	0.1 ± 0	0.1 ± 0	0.1 ± 0	0.1 ± 0	0.1 ± 0
Human	0.3 ± 0	0.2 ± 0	0.2 ± 0	0.2 ± 0	0.1 ± 0	0.2 ± 0	0.1 ± 0	0.1 ± 0	0.1 ± 0
Combined reference	0.3 ± 0	0.2 ± 0	0.2 ± 0	0.1 ± 0	0.1 ± 0	0.1 ± 0	0.1 ± 0	0.1 ± 0	0.1 ± 0

Supplementary Table 3. Percentage of reads mapping to the human genome after removing reads aligning to the human genome with ALN

Seed length	After removing reads (%)	Originally aligned with MEM (%)
18	15.30611	24.1
19	13.27906	22.4
20	11.88606	21.2
21	10.96001	20.4
22	10.41483	19.9
23	10.06839	19.6
24	9.841806	19.4
25	9.661077	19.2
26	9.499251	19.1
27	9.348837	19
28	9.173822	18.8
29	9.035228	18.7
30	8.875695	18.5
40	3.1982	13.1
50	0.975156	10.2
60	0.250332	7.9
70	0.00418	5
72	7.4E-05	4.7
74	0	4.5
75	0	4.4
76	0	4.3
